# Bioreactance-based passive leg raising test can predict fluid responsiveness in patients with sepsis

**DOI:** 10.1186/cc14257

**Published:** 2015-03-16

**Authors:** C Hu, H Tong, G Cai, J Teboul, J Yan, X Lv, Q Xu, J Chen, Q Rao, M Yan

**Affiliations:** 1 Zhejiang Hospital, Hangzhou, China; 2 Bicetre Hospital - University Paris-South, Paris, France

## Introduction

Fluid administration is always important and difficult during the therapy of patients with sepsis. Accurately predicting fluid responsiveness and thus estimating whether the patient will benefit from fluid therapy seems particularly important. The present study intended to predict fluid responsiveness in patients with sepsis using a bioreactance-based passive leg raising test, and to compare this approach with the commonly used central venous pressure (CVP) approach.

## Methods

This prospective, single-center study included 80 patients with sepsis from the Department of Critical Care Medicine of Zhejiang Hospital. Patients were randomly assigned to either Group A or Group B, with patients of in Group A first taking the passive leg raising test and then taking the fluid infusion test, while patients in Group B followed the opposite protocol. NICOM was used to continuously record hemodynamic parameters such as cardiac output (CO), heart rate (HR) and central venous pressure (CVP), at baseline1, PLR, baseline2, and volume expansion (VE). Fluid responsiveness was defined as the change in CO (ΔCO) ≥10% after VE.

## Results

CO increased during PLR (from 5.21 ± 2.34 to 6.03 ± 2.73 l/ minute, *P *< 0.05); and after VE (from 5.09 ± 1.99 to 5.60 ± 2.11 l/minute, *P *< 0.05). The PLR-induced change in CO (ΔCOPLR) and the VE-induced change in CO (ΔCOVE) were highly correlated (*r *= 0.80 (0.64 to 0.90)), while the CVP and ΔCOVE were uncorrelated (*r *= 0.12 (-0.16 to 0.32)). The areas under the ROC curves of ΔCOPLR and ΔCVP for predicting fluid responsiveness were 0.868 and 0.514 respectively. ΔCOPLR ≥10% was found to predict fluid responsiveness with a sensitivity of 86% and a specificity of 79%.

## Conclusion

Bioreactance-based PLR could predict fluid responsiveness in patients with sepsis, while CVP could not.
